# The Microplastics Iceberg: Filling Gaps in Our Understanding

**DOI:** 10.3390/polym15163356

**Published:** 2023-08-10

**Authors:** Diana Rede, Cristina Delerue-Matos, Virgínia Cruz Fernandes

**Affiliations:** 1 REQUIMTE/LAQV, Instituto Superior de Engenharia do Porto, Instituto Politécnico do Porto, Rua Dr. António Bernardino de Almeida, 431, 4249-015 Porto, Portugal; dsgmr@isep.ipp.pt (D.R.); cmm@isep.ipp.pt (C.D.-M.); 2 Departamento de Química, Faculdade de Ciências, Universidade do Porto, rua do Campo Alegre s/n, 4169-007 Porto, Portugal

**Keywords:** plastic pollution, emerging contaminants, analytical methods, ecotoxicity, oxidative stress

## Abstract

Plastic is an indispensable material in modern society; however, high production rates combined with inadequate waste management and disposal have resulted in enormous stress on ecosystems. In addition, plastics can become smaller particles known as microplastics (MPs) due to physical, chemical, and biological drivers. MP pollution has become a significant environmental problem affecting terrestrial and aquatic ecosystems worldwide. Although the topic is not entirely new, it is of great importance to the field of polymers, drawing attention to specific gaps in the existing literature, identifying future areas of research, and improving the understanding of MP pollution and its environmental impacts. Despite progress in this field, problems remain. The lack of standardized methods for MP sampling, separation, extraction, and detection makes it difficult to collect information and establish links between studies. In addition, the distribution and pathways of MPs in ecosystems remain unknown because of their heterogeneous nature and the complex matrices in which they occur. Second, toxicological tests showed that MPs can be ingested by a wide range of organisms, such as *Danio rerio* and *Eisenia fetida,* resulting in gut obstruction, physical damage, histological changes, and oxidative stress. The uptake of MP and their toxicological effects depend on their shape, size, concentration, and polymer composition. Furthermore, MPs can enter the food chain, raising concerns regarding potential contaminations for human and environmental health. This review paper sheds light on the pressing issue of MP pollution and highlights the need for interdisciplinary collaboration between scientists, policymakers, and industry leaders.

## 1. Introduction

Plastic debris and microplastics (MPs) (defined as plastic particles <5 mm) have been widely found in a variety of environmental media, such as air, water, and soil, and their persistence and complexity are considered major global concerns [1,2]. MPs are classified as primary if they are industrially produced, or secondary if they are derived from the fragmentation of larger plastic items [3,4]. Owing to the widespread use and disposal of plastics, as well as the heterogeneity of polymers, the sources of MPs in the environment and the routes of contamination can be difficult to determine. Land-based activities are considered the primary source of MPs, but ocean-related activities such as fishing and shipping can also contribute [5,6]. Typically, MPs tend to be more prevalent in urbanized areas and regions with ineffective waste management, although they can also be dispersed by the airstream and deposited across water and land [7,8,9]. Information on the presence, fate, and long-term effects of MPs on ecosystems (particularly in freshwater and terrestrial environments) is scarce. Indeed, there is a lack of standardized sampling, pretreatment, extraction, and analysis methods for MPs, making comparisons between studies quite challenging [2,4]. There is growing evidence suggesting that MPs can have negative effects on individual organisms [10,11,12,13,14,15,16,17]; for instance, MPs ingested by zebrafish (*Danio rerio*) can accumulate in the gut, causing inflammation and oxidative stress in tissues [13]. 

Research and development of effective methods to detect and monitor MPs in various environmental media is essential because of the significant environmental impact and potential health risks associated with them. The standardization of analytical methods would provide more reliable and robust data, which in turn would facilitate the development of effective regulations to mitigate the impact of MPs, promote the use of alternative materials to plastics, and establish defensible waste management practices. This study provides an integrated literature review on pollution from MPs, focusing on an analytical methodology. The study covers a wide range of studies that examined the sources and transport of MPs into the environment, as well as the concentrations and toxicological effects of MP exposure. It includes both research and reviews that address advances in extraction and detection methods, quantification techniques, and innovations to identify MPs in various environmental matrices while analyzing the pathways of entry involved. To prepare this review, relevant studies were retrieved from the ScienceDirect database covering the period from 2017 to 2023. By summarizing and reviewing the literature on these critical aspects, this review aims to contribute to a deeper understanding of the environmental pollution of MP and to promote progress in the field of analytical methods for their study.

## 2. Emerging Contamination: From Plastic to Microplastics

Plastics comprise a wide variety of materials that are produced from different sources, namely fossil origins (i.e., crude oil, gas, etc.), renewable (e.g., sugar cane, vegetable oils, etc.), or mineral base (i.e., salt) [18]. Large-scale plastic production began in the early 1950s and products made from these materials are all around playing a fundamental role in a myriad of sectors, such as packaging, construction, electronics, agriculture, textile, cosmetics, among others [19,20,21]. The use of plastics deeply shapes the development of modern society as they bring safety, hygiene, comfort, and well-being [18,20]. Many types of chemical additives are used in plastic production to improve its properties, including plasticizers, stabilizers, flame retardants, and colorants [22,23,24]. It is important to note that the characteristics that make plastic materials desirable (hydrophobicity, durability, etc.) are those that make them persistent and prevalent in terrestrial, freshwater, estuarine, coastal, and marine environments [2,25].

In 2021, global plastic production reached 390.7 million tonnes, with Asia being responsible for half of the production followed by North America and Europe. Packaging, building and construction, and the automotive sector were the three biggest end-use markets, with 44%, 18%, and 8% of the consumption, respectively [26]. Fossil-based resins represented 90.2%, and the most produced polymers were polypropylene (PP) (19.3%), low-density polyethylene (PE-LD) (14.4%), polyvinyl-chloride (PVC) (12.9%), high-density polyethylene (PE-HD) (12.5%), polyethylene terephthalate (PET) (6.2%), polyurethane (PUR) (5.5%), and polystyrene (PS) (5.3%). Circular plastics (i.e., recycled and biobased/bioattributed plastics) represent approximately 9.8% of the global production [26]. It was estimated that in 2015, around 8.3 billion tonnes of plastics were produced worldwide, of which 6.3 billion tonnes ended up as waste with 9% being recycled, 12% energetically recovered, and 79% deposited in landfills or leaked out of formal waste collection systems accumulating in terrestrial or marine environments (i.e., single-use packaging) [21,27,28]. According to Plastics Europe, the separate waste collection enables recycling rates 13 times higher than mixed waste collection systems [26].

**Figure 1:**
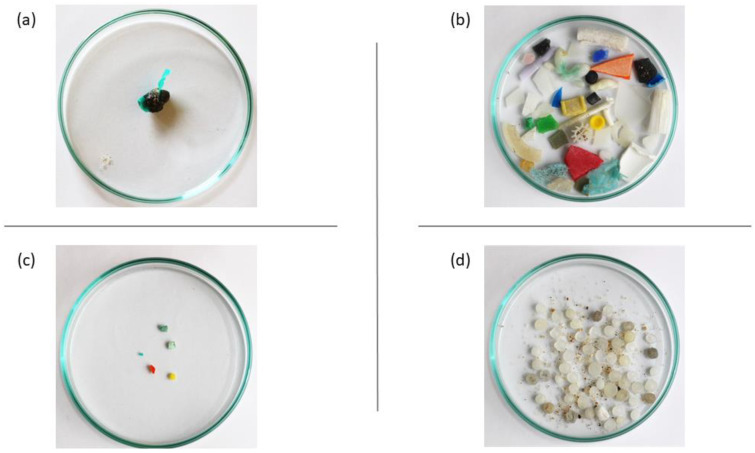
Types of plastic debris found on a beach in Northern Portugal. The plastic materials include: (**a**) a plastiglomerate, (**b**) mesoplastic fragments, (**c**,**d**) microplastics.

The different physical properties of each polymer may affect its behavior in the environment. For instance, in the aquatic environment, the tendency of particles to float or settle in sediments is usually related to the density of the polymer [2,9]. However, buoyant particles of PP and PE can sink and be retained within sediments, which means that even when physical properties are well known, predicting the fate of polymers in the environment is very challenging [2,29]. From sediments in deep seas to underwater canyon, encapsulated in Arctic sea ice, or massive accumulations at sea surface waters, such as *the Great Pacific Garbage Patch*, plastic materials have been identified all across the globe [29,30,31,32]. Moreover, the ability of plastics to fuse with volcanic rocks, sediments, and organic materials may lead to the formation of solid rocklike structures, the *plastiglomerates* (Figure 1a) [33,34]. Furthermore, it has been shown that chemical additives can leach out during the life cycle of the product [22,35,36]. At the same time, due to the hydrophobic surface of plastic materials, they can adsorb other chemical contaminants (e.g., metals, polychlorinated biphenyls, polycyclic aromatic hydrocarbons, and/or pesticides) [22,35,36,37]. Synergistic interactions may occur between them, resulting in an enhancement of the toxicity in living organisms [22,35,36,37,38]. It is possible that if plastic particles were taken up by biota, they could act as carriers for other contaminants along the food chain—a *Trojan horse effect* [3,37,39,40,41].

Plastic litter can be degraded by physical, chemical, and biological drivers (i.e., ultraviolet radiation, wind or water erosion, etc.) and become smaller pieces, usually classified by size, namely, megaplastics (>100 mm), macroplastics (>20 mm), mesoplastics (20–5 mm, Figure 1b)), MPs (<5 mm, Figure 1c,d), and nanoplastics (NPs) (1 to 1000 nm), although the adopted terminology can vary [7,39,42,43,44]. Plastic debris was pointed out as a serious environmental issue in the 1970s, though only in 2004 was the term microplastic applied for the first time [9,45]. Currently, environmental contamination caused by MPs is in the public domain, being considered an emerging issue of global interest [16,46,47]. MPs fall into two groups: primary MPs if they are produced for industrial purposes, such as PE microbeads used as exfoliating agents in personal care products; or secondary MPs if they are derived from the fragmentation of larger items [6,39,40]. Secondary MPs correspond to the majority of MPs that can be found in the natural environment [3,48].

Owing to the massive production of plastic materials and the ineffectiveness of waste management and disposal, there is no question about the challenge that plastic pollution represents in our society [36]. The wide variability of plastic types and sizes has hindered the development of standardized extraction and detection methods for MPs in environmental samples [35,49]. There has been an increase in the development of programs and guidelines for assessing marine litter and in the number of studies on the fate and effects of marine compartments [8,45]. However, sources, in situ formation, distribution, transport pathways, interactions, and ecotoxicological effects of MPs and other chemical contaminants on the environment, particularly in terrestrial and freshwater compartments, have not received sufficient attention, and the data are scattered [39,45]. Both have been seen mainly as sinks and transport routes for MPs to reach the seas and oceans [2,6]. Monitoring programs are generally based on the measurement of chemical concentrations in different compartments. They do not provide useful information on real impacts on organisms and ecosystems, making it difficult to prepare protection and mitigation plans [36,39]. In Europe, approximately 26 million tonnes of plastic waste are produced annually [50]. To tackle plastic pollution and marine litter and to accelerate the transition to a resource-efficient circular plastic economy, the European Commission has established the European Plastics Strategy as part of the Circular Economy Action Plan and the Framework on Biobased, Biodegradable, and Compostable Plastics [50,51]. It brings clarity to consumers and the industry on single-use plastics, plastic packaging, MPs, and circular plastics. It is estimated that between 75,000 and 300,000 tonnes of MPs are released into the environment every year in the EU [50]. While there is still no law in place for MPs, the European Chemical Agency has put forward a proposal for a restriction on MPs intentionally added to mixtures, which is currently under discussion with member state authorities and voting. If adopted, this restriction would reduce the quantity of MPs released to approximately 500,000 tonnes over 20 years [52].

## 3. The Ubiquity of Microplastics in the Environment: Input Pathways and Transport

**Figure 2:**
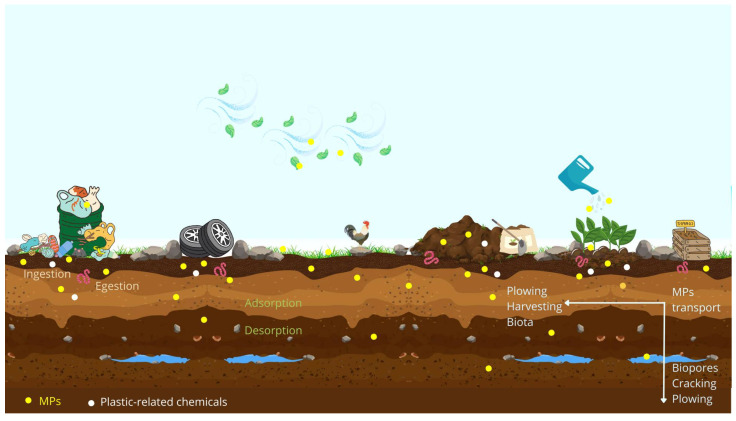
Sources and routes of microplastics and plastic-related chemical pollution in soil environment.

The sources of MPs in the environment can be numerous, and the routes of contamination can be difficult to define because of the massive plastic production and consumption (especially single-use packaging), the leakage of plastic from waste streams, and the heterogeneity of polymers [2,6,53]. Land-based activities are pointed out as the main sources of MPs, and as environmental compartments are linked, it is expected that contaminants migrate between them (Figure 2) [5,6]. Moreover, ocean-based activities, such as fishing, aquaculture, and merchant ships, might also release MPs directly into the environment, either by the accidental loss and fragmentation of plastic materials or by illegal dumping of plastic waste at sea [6,41,53]. MPs are likely to be more abundant in urbanized areas as well as in areas where waste management programs are ineffective [2,22,54]. Additionally, lighter plastic items from construction materials, artificial turf, and household dust can be transported by wind action and deposited by atmospheric fallout across water bodies and/or land [8,9,30].

Industrial complexes might contribute to the input of primary MPs into the environment, mostly due to improper handling during production, packaging, and transportation [25,37]. Some examples are plastic resin pellets (commonly known as *nurdles*, Figure 1d), flakes, or plastic powder, used as raw materials in the production of larger items, waste from plastic production, regranulate from plastic recycling, and products containing abrasives [37]. Furthermore, wastewater treatment plants (WWTPs) are thought to be an important land-based source of primary MPs, namely plastic particles from personal care products (i.e., hand cleaners or toothpaste, to name a few) and medical products [3,22,25,55]. In addition, wastewater may contain secondary synthetic microfibers from laundry (i.e., PA), and other plastic debris that, due to inadequate disposition, end up in wastewater streams [2]. The resulting sewage sludge is often disposed of in soil or converted into compost or biosolids (pasteurized sewage sludge) and applied as fertilizer (Figure 2) [25,28,55]. Regarding the effluents of WWTPs, they can be discharged into the aquatic environment or be used as reclaimed water and are frequently seen as a major source of MPs [3,8,25,39,56].

Approximately 95% of MPs captured by WWTPs are retained in the sludge phase [8,55,57]. Every day, large amounts of sewage sludge are produced and widely applied to the soil for agricultural purposes [57,58]. Considering the properties of polymers, it is expected that MPs will remain barely unchanged over time [2,25,59]. For example, the original properties of microfibers, which are considered the most abundant microparticles in natural environments, have been preserved for 15 years after biosolid application [22]. The remaining 5% of MPs that pass through WWTPs are directly and continuously released into aquatic systems [8,25]. As MPs can be retained within sediments and buried close to the outlet of treated wastewater, not all plastic particles discharged into freshwater are transported to marine environments [22,60]. For instance, in industrialized areas, rivers may have higher concentrations of MPs than marine environments [22]. Furthermore, when modern WWTP facilities are not available or in the case of an overflow, wastewater is directly input into aquatic systems without any treatment [2,9]. Nonetheless, it is undeniable that rivers and freshwater bodies are the major carriers of MPs from land to oceans [6,8]. According to Zhang and Liu, only 1% of global plastic waste is directly input into the marine environment, which means that the largest amount is transferred from freshwater and soil [28].

A direct source of secondary MPs is the fragmentation of meso- and macroplastic litter already present in the environment [1,2]. Physical abrasion and ultraviolet radiation are considered to be the driving forces that trigger the fragmentation of plastic items by altering their chemical, physical, and mechanical properties [9,61,62]. Another source of secondary MPs is living organisms that mistake plastic for food [63,64]. For example, caddisfly larvae use external feeding appendages to actively fragment and physically alter plastics [63]. Similarly, earthworms contribute to the biofragmentation of plastic litter, as reported by Kwak and An [64]. In addition, landfills can facilitate the entry of MPs into the environment, either through the loss of plastic materials during waste collection or mismanagement [6,25,54]. MPs can also accumulate in the environment through the fragmentation of tire wear particles, road-marking paints, and particles derived from vehicle components. Tire abrasion particles may be introduced into roadside environments via dust or wash-off, decreasing plant growth even at low concentrations, altering bulk density and soil aeration, and ultimately the biogeochemical cycling [39,54,65,66].

The plastic mulching used in agriculture promotes the incorporation of MPs into the environment [25,39,54]. This widespread agricultural technique contributes to improve production, but plastic-related chemicals such as phthalates (plastic plasticizers) can be released into crops along with MPs [39,54]. Moreover, it is expected that dense polymers remain in the soil and are transported into deeper soil layers by the action of biota, harvesting, ploughing, soil cracking, and wet–dry cycles [64,67,68]. On the other hand, lighter polymers are more likely to be transported across water bodies or land, by the action of wind and water (Figure 2) [8,9]. It is important to note that plastic debris is not only transported by rivers from land to the ocean; plastic litter in the aquatic environment can return to land due to high tides or floods, reinforcing the idea that pollutants drift between environmental compartments [2,30,39].

## 4. Unveiling Microplastics in the Environment: Advances and Challenges in Analytics

MPs have been reported worldwide from the water and sediments of Hiroshima Bay (Japan), amended agricultural soil in Mellipilla (Chile), and snow from the Alps (Europe) [30,69,70,71]. From an analytical point of view, some challenges can be identified, such as the lack of reference material needed for method validation and the inexistence of standardized analysis methods that allow the accurate comparison of results [55,72]. As shown in Table 1, there are differences in MP extraction procedures between the same sample types, such as agricultural soils from the Chai River valley (China) [28] and Middle Franconia (Germany) [58] or freshwater from Lakee Saimaa (Finland) [73] and Lakee Winnipeg (Canada) [3]. Furthermore, analytical methodologies designed for the extraction of MPs from aqueous samples have been adapted to solid samples, such as soil. However, the high complexity of solid matrices, especially if they are rich in organic matter, and the presence of other chemical contaminants (e.g., PAHs) hinder the extraction and identification of MPs [55,74]. Moreover, the degradation of plastics under natural conditions causes changes in the surface and functional groups. This means that depending on the degree of aging, MPs of the same polymer can exhibit different thermochemical properties, which can decrease the accuracy of the analysis [62,75,76,77]. Concerns regarding cross-contamination have been highlighted and the impossibility of working in a plastic-free environment has been recognized [56,78,79]. It is recommended that the use of plastic materials be avoided and limited as far as possible; however, in some cases, it cannot be eliminated [56]. However, as highlighted by Miller et al. (2021), there is a perceived need for the further development of rigorous study designs in MP research, with relatively few studies currently incorporating comprehensive quality assurance and quality control (QA/QC) measures commonly found in more established fields of trace chemical contaminant monitoring. The pressing need in this area is to establish standardized methods for QA/QC, which should include the systematic collection and reporting of field and laboratory blanks, as well as a careful consideration of background contamination in field samples [80]. 

**Figure 3:**
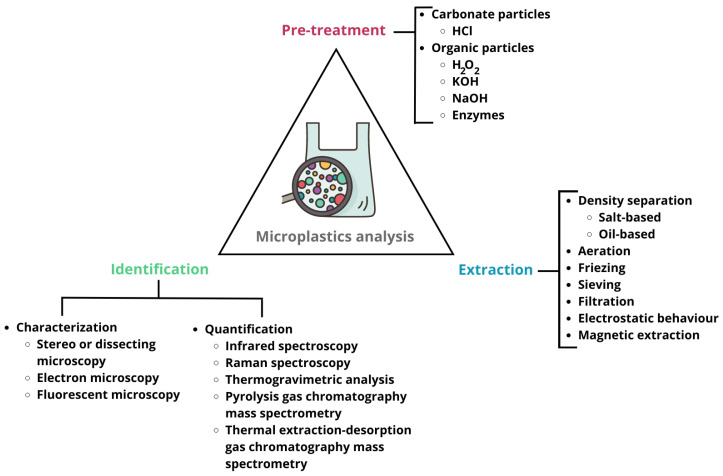
The main steps involved in analysing microplastics in environmental samples: pretreatment, extraction, and identification.

In general, the methods reported in the literature are carried out in three main steps: sample pretreatment (e.g., drying, size fractioning, and chemical digestion of organic matter), MP extraction (e.g., sieving or density separation), and the identification of the recovered MPs (Figure 3). The presence of nontarget impurities, such as dust or organic matter, can bias MP identification, and it is more challenging to separate and characterize MPs from samples with a high organic matter content such as soil than from water matrices [54,77]. In fact, the elimination of organic matter is a sore point in sample pretreatment, since it is necessary to avoid or limit the possible damage that oxidizing agents, usually hydrogen peroxide (H_2_O_2_) [8,78,81,82] or Fenton’s reagent [3,28,73,78], may cause in MP particles [3,22,56,81]. For instance, a method developed by Li et al. (2019) for analyzing MPs in soil and sludge samples comprised two digestions to remove organic matter: a first with 30% H_2_O_2_ at 70 °C and a second with H_2_O_2_ and sulfuric acid at 70 °C. The authors observed a slight dissolution of PA, possibly owing to the last step of the process [81]. As an alternative to chemical oxidation, Mintenig et al. (2017) developed a greener, multistep, plastic-preserving enzymatic-oxidative method to degrade the organic matter of wastewater from Lower Saxony (Germany), but the process takes 20 days (incubation, 24 h; protease, 48 h; lipase, 96 h; cellulase, 6 d), which is time consuming [56]. 

Salt-based density separation followed by filtration is the most widely used procedure for extracting MPs [8,28,56,57,69,70,81,83]. However, it is not always easy to float the densest polymers, which can undermine the reliability of the results, because high-density MPs may be underestimated or not reported. To overcome these difficulties, some authors have proposed an oil-based density separation by exploiting the oleophilic properties of plastic materials. Using this technique, it is necessary to rinse the sample with organic solvents (e.g., n-hexane) after extraction to reduce the interference of oil in the identification analysis [84,85,86,87]. Furthermore, aeration and freezing have been used as adjuncts to the density separation process [84,88,89]. However, it is difficult to avoid disturbances in the settled solid phase during filtration when using beakers and clogging when using separation funnels. In addition, laboratory glassware can pose a challenge, as some MPs adhere to glass walls, and in the case of solid samples, more than one filter can be generated per sample [90,91]. To mitigate sample loss caused by repeated filtering or sieving, some authors have developed custom-made labware and devices utilizing density separation/flotation or sieving techniques. These include a small stainless-steel sieve that fits into a 200 mL glass beaker [90], a PTFE cylinder equipped with removable caps and a piston [84], a spiral conveyor operated within a glass separation funnel [92], the Munich Plastic Sediment Separator (MPSS) [93], the Sediment-microplastic Isolation (SMI) [94], and the JAMSTEC Microplastic-sediment Separator (JAMSS) [91]. In 2012, a team of researchers developed the MPSS, a stainless-steel device standing about 1.75 m tall, that uses zinc chloride as a flotation medium. The study reported recovery rates of 100% for larger MPs (1–5 mm) and 95.5% for MPs smaller than 1 mm in spiked and organic matter-free sediments. Zobkov and Esiukova later re-evaluated the extraction efficiencies of MPSS using unprepared sediment samples collected from the Baltic Sea, obtaining results between 13 and 39% [93]. SMI, a portable plastic device, is based on the MPSS’s design and is compatible with both coarse and fine beach sediments. It can extract MPs with an average recovery rate of 95.8% in a single step [94]. JAMSS consists of two modified glass plates, with the upper plate attached to an open glass tube and the lower plate attached to a cylindrical glass container [91]. Devices with different sample-processing capabilities can be assembled by modifying the volume of the tube/container. JAMSS is used in combination with a sodium iodine solution and prevents the resuspension of decanted sediments into the supernatant, with recovery rates ranging from 94 to 98% in a single-step extraction [91].

Magnetic and electrostatic techniques have been tested as alternatives to density-based methods for extracting MPs from environmental samples. The aim is to reduce sample loss, avoid toxic salts, improve high-density polymer extraction, and accelerate the experimental procedure [95,96,97,98]. One such technique is magnetic seeded filtration (MSF), which uses seed particles to agglomerate with micro- and nanoscale particles, thereby enabling magnetic separation [95]. Using MSF, Rhein et al. achieved a 95% separation efficiency of MPs from dilute suspensions across a wide range of pH values [95]. Both magnetic and nonmagnetic fractions can be retrieved by the chemical breakup of agglomerates [99]. Moreover, using hydrophobic seed particles, the MSF approach is a promising purification step that may aid in quantifying MP extracted from complex matrices, such as sewage sludge [100]. Hydrophobic iron nanoparticles were tested by Grbic et al. in seawater spiked with PE, PS, PET, PUR, PVC, and PP MPs with recovery rates higher than 90% [96]. The authors applied this technique to extract MPs from freshwater and sediments and recovered 84% and 78% of the MPs, respectively. The adsorption efficiency of modified iron nanoparticles depends on the hydrophobicity and crystallinity of MPs [101]. Furthermore, the presence of organic and inorganic particles in the media hinders the ability of the iron nanoparticles to contact the MPs, which may account for the decreased extraction efficiency observed in sediments [96,98]. Additionally, the extraction process results in the fragmentation of brittle MPs. Moreover, Ramage et al. employed hydrophobized iron nanoparticles along with the high-gradient magnetic separation (HGMS) technique for the extraction of MPs from four soil types, namely, loam, high-carbon loamy sand, sandy loam, and high-clay sandy loam [98]. By utilizing an electromagnetic field and magnetically susceptible wires, HGMS systems can produce steep gradients that result in a net force on the particles, causing their separation via competition between magnetic and gravity settling forces. Magnetic recovery is determined by the ability of the modified iron nanoparticles to bind to the MP surface and particle size, which implies that the polymer type has no impact on it. To implement this method in soils, it is necessary to first extract paramagnetic minerals naturally present in the sample matrix to prevent wire blockage; however, MPs aggregated into clay can also be removed. This technique enables the processing of each sample within 30 min, which marks a significant reduction from the 2–3 days required by the density separation method. The recovery rate obtained (>88%) is dependent on several factors, including MPs’ size, soil mineralogy, and carbon content [98]. Regarding electrostatic separation, its application has been limited to beach sand and river sediments [102,103,104]. This technology is used for metal/plastic waste separation and mineral refinement, and it utilizes a dry separation process to segregate metal particles with a diameter <8 mm from nonconductive materials such as commonly used plastics [102,103]. A scaled-down version of the Korona–Walzen–Scheider electrostatic metal separator system, which is widely used in the recycling industry, has been used to gain insights into the potential application of an electrostatic separation of MPs with diverse characteristics, including density, size, shape, and age [102,103,104]. Although its application has been limited to sediment testing, it was observed that the recovery of the method was highly dependent on the size of MP particles and sample composition and complexity, namely mineral and organic matter contents [103]. Thus, all the aforementioned procedures can be less efficient and/or more laborious for soil or sludge samples [73].

MPs’ identification typically involves two types of analytical techniques: particle-based methods such as microscopy, Fourier transform infrared (FT-IR), or Raman spectroscopy, and mass-based determination methods, such as thermal desorption gas chromatography coupled with mass spectrometry (TED-GC-MS) [104]. Despite the need for further research, there is an increasing concern among politicians and scientists regarding the lack of reliable and comparable data on environmental MP pollution. A survey of existing papers on the study of MPs using FT-IR spectroscopy revealed a lack of information about the experimental setup, which is crucial for scientific reproducibility, indicating poor quality criteria and a lack of control samples and validation in analyses [105]. Moreover, there are currently no established units for reporting MP concentrations. Table 1 displays varying units of MPs concentration reported in studies, such as particles per liter [78], particles per kilogram [69], particles per 50 g dry weight [83], particles per gram [70,73], particles per 50 kg dry weight [58], particles per cubic meter [56], particles per square kilometer [3], and newtons per liter [30]. If studies do not provide the information needed to compute the unit conversion, it becomes difficult to compare the results and draw conclusions [2,22]. 

Certain studies solely relied on optical microscopy techniques (dissecting microscopes or stereomicroscopes) to quantify MPs in various environmental samples such as agricultural soil, beach sand, or amended agricultural soil [28,70,83]. No other methods were used to validate the results or identify the types of polymers detected. Naked eye detection can only identify visible particles, but with the use of optical microscopes, particles larger than 0.42 µm can potentially be detected [2,22]. However, visual selection has limitations in terms of accuracy and precision, as it can be difficult to distinguish MPs from other materials, leading to the overestimation of microparticle numbers [22,39,81]. To overcome this, other analytical techniques based on molecular “fingerprint” for identifying polymers, such as FT-IR (including micro-FT-IR or attenuated total reflectance (ATR)-FT-IR) or Raman spectroscopy, have commonly been used, with FT-IR being particularly prevalent. FT-IR enables the detection of MPs as small as <20 μm, whereas Raman spectroscopy can identify MPs as small as <1 μm [2,22]. However, it is worth noting that the irregular surfaces of some polymer particles may interfere with the identification using FT-IR spectroscopy, and the presence of additives such as pigments or plasticizers may interfere with the identification using Raman spectroscopy [106]. Additionally, a sample analysis may require a significant amount of time, ranging from several hours to several days [105]. Mass-based methods facilitate expeditious data acquisition on the types and quantities of polymers in environmental samples. Moreover, these methods entail reduced contamination risks compared with particle-based methods, as they are less susceptible to atmospheric exposure [104]. However, these methods are destructive, which means that information on particle count, size, and shape characteristics cannot be obtained. Examples of mass-determining methods include thermogravimetric analysis (TGA), TED-GC-MS, and pyrolysis in combination with gas chromatography and mass spectrometry (Py-GC-MS) [76,86,107,108,109,110,111]. The application of headspace solid-phase microextraction coupled with gas chromatography and mass spectrometry (HS-SPME-GC-MS) and liquid chromatography with UV detection (LC-UV) had also been explored [87,109,110] Chromatographic techniques enable the determination of monomers as well as plastic additives or chemicals that have been sorbed onto the surface of MPs, such as pesticides or pharmaceuticals. Other relevant techniques, such as Nile red staining combined with fluorescent microscopy, scanning electron microscopy (SEM) (including field emission (FE)-SEM and SEM–energy dispersive X-ray spectroscopy (EDX)), X-ray photoelectron spectroscopy (XPS), differential scanning calorimetry (DSC), and nuclear magnetic resonance (NMR) spectroscopy have been used to obtain information on size, morphology, behavior, and type of MPs [69,78,103,104,111,112,113].

In summary, the importance of harmonizing standardized analytical methods has been highlighted in numerous studies [104,112]. Additionally, the absence of standardized practices hinders accurate occurrence calculations and makes cross-study comparisons challenging. By understanding the sources of background contamination, researchers can better select appropriate reporting methods and implement additional measures to minimize their impact. Adopting standardized QA/QC procedures is crucial to ensure the accuracy and reliability of MP data, enabling meaningful comparisons between different research studies [80]. Although there may be limited knowledge on the subject, the dedication of the scientific community to the development of reliable analytical methods is evident from the increasing number of recent studies and proposed optimizations. Obviously, all methods have strengths and weaknesses, and their usage should be tailored to align with the research objectives of each study, as they can be complementary or coupled [104,112]. For instance, when using Nile red staining methods, nonplastic hydrophobic particles can be dyed, requiring confirmation via spectroscopic techniques to avoid any overestimation of MPs. Another example is Raman microscopy, which enables the determination of the polymer type, as well as the observation of the morphology, size, and color.

**Table 1:** Summary of MPs’ concentrations in the environment covering a range of compartments. The selected studies had methodological details. (dw: dry weight; WTP: water treatment plant; KWS: Korona–Walzen–Scheider; Py-GC-MS: pyrolysis gas chromatography–mass spectrometry; DSC: differential scanning calorimetry; FT-IR: Fourier transform infrared spectroscopy; μ-FT-IR: micro-FTIR; ATR-FT-IR: attenuated total reflection FTIR; FE-SEM: field-emission scanning electron microscopy; SM-EDX: SEM energy electron microscopy; X-ray CT: X-ray computed tomography; HS-SPME-GC-MS: headspace solid-phase microextraction coupled with gas chromatography and mass spectrometry; LC-UV: liquid chromatography with UV detection; TED-GC-MS: thermal desorption gas chromatography–mass spectrometry; TGA-MS: thermogravimetry–mass spectrometry;. EGA-MS: evolved gas analysis–mass spectrometry).

Sample Type	Location	Study Findings	Pretreatment	Extraction and Identification Techniques	Ref.
Beach sand	The Netherlands	Intertidal zone: 29.10 ± 17.75 MPs per 50 g dw High tidal line: 25.20 ± 12.37 MPS per 50 g dw Supralittoral zone (30 m from the dunes): 21.30 ± 3.62 MPs per 50 g dw Supralittoral zone (10 m from the dunes): 25.90 ± 10.91 MPS per 50 g dw	Samples were dried (48 h, 75 °C), and stored at room temperature for extraction.	Samples were floated in NaCl solution (358.9 g L^−1^). After mixing for 2 min at 600 rpm, the mixture settled for 6 h, then the supernatant was filtered. MPs were counted on filter papers using a stereomicroscope. NaCl flotation was used.	[83]
Quartz sand Freshwater sediment Beach sand	Germany	99% of the original sample mass was removed without loss of MPs. 150 g quartz sand reduced to 2.34 ± 0.17 g. 150 g freshwater sediment reduced to 2.33 ± 0.13 g. 150 g beach sand reduced to 2.00 ± 0.04 g. 150 g particulate matter reduced to 2.51 ± 0.23 g.	Sample was sieved through 20, 63, 200, 630, and 2000 mm sieves. 20 g of freeze-dried material, 100 mL of bidistilled water, and agate balls were added to each sieve, that was sonicated for 1 min, and sieved manually. The procedure was repeated 10 times and the remaining fractions were centrifuged and dried at 105 °C.	150 g of sample material was spiked with 10 particles of each type of MP. Separation using the KWS electrostatic method, and each fraction was separated 3 times. Samples were digested, subjected to density separation, and characterized by Py-GC-MS.	[102]
Sand River sediment		Mass of sand was reduced by 98% after electrostatic separation. Mass reduction of sediment samples reduced by 70–78% after electrostatic separation, and above 99% after density separation. Recovery of MPs was polymer-specific: PCL: 50 ± 8%–74 ± 9%; LD-PE: 93 ± 9%–114 ± 9%; and PET 82 ± 11%–120 ± 18%.	Samples dried at 60 °C. Particles with diameters >2 mm were removed by sieving. Samples spiked with PCL, LD-PE, and PET (63–200 µm).	Electrostatic separation combined with density separation, identification, and quantification by DSC.	[104]
Estuarine sediments	Japan	Fenton’s reagent affected the size of PE and PET. Recovery rates for density separation using an overflow column with top inflow (OC-T): 90.7% (±7.7% SD) for large MPs (>0.5 mm) and 95.0% (±12.5% SD) for small MPs (<0.5–0.160 mm). 91.7% of stained particles were confirmed as MPs.	2 protocols were tested for organic matter removal: (1) 20 mL of a 30% H_2_O_2_ added to 60 mL of sample; (2) 20 mL of a 0.05 M Fe(II) solution (2.5 g of FeSO_4_.7H_2_O, 165 mL water and 1 mL of concentrated H_2_SO_4_) added to 60 mL of sample. Samples were left to react for 24 h at room temperature and then freeze-dried at 80 °C under vacuum.	Density separation was tested using 4 columns, filled with ZnCl_2_ solution (1.5 g cm^−3^): an SMI unit; a simple decanting column; an OC-T; and an overflow column with mid-level inflow (OC-M). MPs were concentrated on vacuum glass fiber filters and drops of a Nile red solution (1 mg Nile red dye in 1 mL 99.5% acetone, diluted with 100 mL distilled water) were spread. Stained filters were inspected with a binocular microscope. Automated epifluorescence microscopic image analysis of Nile red-stained filters with FT-IR validation for polymer identification.	[111]
Beach sediments		Foamed polystyrene (FPS) (0.3 to <5 mm): 80–17,500 particles m^−2^; 5–1206 pieces kg^−1^ dw PE and PP (0.3 to <5 mm): 0–1640 pieces m^−2^; 0–200 particles kg^−1^ dw	-	Samples floated in saline solution (200 mL:100 mL sediment) for 30 s, settled for 2 min, and then sieved through a 355 μm mesh. The residue was rinsed, vacuum-filtered, and MPs were collected via visual sorting and stereomicroscopy. Identification was done with FT-IR, and structural analysis utilized digital microscopy, FE-SEM, and X-ray CT.	[69]
Bottom sediments		FPS (0.3 to <5 mm): 552–9128 particles m^−2^; 13–221 particles kg^−1^ dw PE and PP (0.3 to <5 mm): 210–1210 particles m^−2^; 5–29 particles kg^−1^ dw	Samples sieved through 5.6 mm and 355 μm mesh.		
Bay surface water		FPS (0.3–<5 mm): 0.004–0.06 particles m^−2^ PE and PP (0.3–<5 mm): 0.03–0.17 particles m^−2^		The residues on the 355 μm mesh were rinsed with distilled water and vacuum-filtered. MPs were collected from samples by visual sorting and stereomicroscopy. Identification was performed using FT-IR and the structure was analyzed by digital microscopy, FE-SEM, and X-ray CT.	
Snow	Arctic and Alps	MPs: 0.02 × 10^3^–154 × 10^3^ N L^−1^ (80% ≤ 25 µm; 98% < 100 µm) Microfibers: 0.043 × 10^3^–10.2 × 10^3^ N L^−1^ (maximum length: 97% < 5 mm; 31% < 500 µm) European fibers were significantly longer than those from Arctic snow.	-	Samples were filtered using aluminum oxide (Al_2_O_3_) filters and dried in a desiccator for 2 days. Particles retained on filters were analyzed by FT-IR imaging.	[30]
Aqueous solution		Linearity (R^2^) > 0.994 Precision: 99.4–99.9%. Limit of detection (LOD): 19–21 μg mL^−1^ Limit of the quantification (LOQ): 74–85 mg mL^−1^	MPs (PE granules < 300 μm, PET fibers 500 μm, and PS beads 0.5–1 mm) dissolved in a deuterated solvent at 50 °C	Samples were analyzed by NMR spectroscopy.	[113]
Freshwater (inlet WTP)	Czech Republic	1473 ± 34–3605 ± 497 particles L^−1^ Common sizes: 1–10 μm Common types: fibers, spherical, and fragments Common polymers: PET and PP	Oxidize each sample with 20 mL of 0.05 M Fe (II) and 20 mL of 30% H_2_O_2_, and stir for 30 min at 75 °C in 250 mL of sample. Allow samples to digest for 24 h.	Samples vacuum-filtered in two-step descending-mesh-size, dry PTFE filters (30 °C, 30 min). Particles retained on filters were analyzed by SEM; particles > 10 μm were analyzed by FT-IR; particles 1–10 μm were analyzed by μ-Raman spectroscopy. Elemental analysis was performed on selected particles using SEM-EDX.	[78]
Drinking water (outlet WTP)		338 ± 76–628 ± 28 particles L^−1^ Common sizes: 1–10 μm Common types: fibers, spherical, and fragments Common polymers: PET PP and polyacrylamide			
Wastewater (effluent)	Germany	MP > 500 µm: 0 × 10^1^–4 × 10^1^ particles m^−3^ MP < 500 µm: 8 × 10^1^–9 × 10^3^ particles m^−3^ Common polymers: PE (59%) and PP (16%)	Samples were incubated at 70 °C for 24 h, then treated with enzymes (protease at 50 °C for 48 h, lipase at 40 °C for 96 h, and cellulase at 50 °C for 6 days). Finally, each sample was filtered through a 500 μm PA net.	<500 μm fractions were filtered with 10 μm stainless-steel screens, and then treated with H_2_O_2_ (35%) at 37 °C for 48 h, followed by incubation at 50 °C for 24 h. MPs were identified using ATR-FT-IR and μ-FT-IR spectroscopy techniques.	[56]
Sewage sludge		MP > 500 µm: not detected MP < 500 µm: 1 × 10^3^–2.4 × 10^4^ particles kg^−1^ dw	125 g of each sample was diluted in 825 deionized water and then mixed with 400 g solid NaOH (24 h at 60 °C). Samples were neutralized with HCl.	Flotation with NaCl (1.14 g cm^−3^). After 96 h, supernatants were rinsed over a 500 μm PA net. Residues were visually inspected using an optical microscope and identification was carried out using ATR-FT-IR. Aliquots of fraction <500 μm filtered using 0.2 µm Al_2_O_3_ filters and analyzed by μ-FT-IR.	
Lake water	Canada	53,000–748,000 particles km^−2^ Common types: fibers (90%), films, and foam	Samples were filtered through a 250 μm mesh sieve and rinsed with deionized water. Each 250 mL sample was oxidized with 20 mL of Fe (II) (0.05 M) and 20 mL of 30% H_2_O_2_ while stirring for 30 min at 75 °C. Samples digested for 24 h.	Each sample was filtered through a 250 μm sieve. MPs were visually identified using a dissecting microscope and SEM-EDX.	[3]
Lake water	Finland	Total MP: 0.3 ± 0.1 particles L^−1^ Microfibers: 0.2 ± 0.1 particles L^−1^	Samples were sieved and dried (40 h, 75 °C). Dried samples were oxidized with 20 mL of Fe (II) (0.05 M) and 60 mL of H_2_O_2_ (30%). Samples settled for 5 min, then were shaken and heated up to 75 °C.	Samples were vacuum-filtrated, and the glass filters were left to dry for 24 h at room temperature. Identification was performed by optical microscopy, FT-IR, and Raman spectroscopy.	[73]
Wastewater (influent)		Total MP: 56.7 ± 12.4 particles L^−1^ Microfibers: 52.6 ± 11.3 particles L^−1^	Samples were sieved and dried (40 h, 75 °C). Dried samples were oxidized with 20 mL of Fe (II) (0.05 M) and 20 mL of H_2_O_2_ (30%). Samples settled for 5 min, then were agitated and heated up to 75 °C.		
Sewage sludge		Activated sludge: 23 ± 4.2 particles g^−1^ dw microfibers: 21.7 ± 4.6 particles g^−1^ Digested sludge: 170.9 ± 28.7 particles g^−1^ dw microfibers: 161.0 ± 25.5 particles g^−1^	Samples were stirred and subsamples (0.1 g dw) were dried at 45 °C for1 8 h.	Identification was performed by optical microscopy, FT-IR and Raman spectroscopy.	
Wastewater (influent)	Spain	Primary effluent (451 ± 106 particles L^−1^) Fragment length: 53–2100 µm Fiber length: 104–4000 µm Secondary effluent (26 ± 14 particles L^−1^) Fragment length: 41–2890 µm Fiber length: 144–1824 µm	Filtration through 3 stainless steel meshes (375, 104 and 25 μm). Filters were exposed to H_2_O_2_ (33%) at 50 °C. After 20–24 h, the filters were rinsed with deionized water.	NaCl flotation was conducted, and samples were stirred for 24 h followed by settling for 24 h. Both supernatant and sediment were then filtered. Identification was performed using stereomicroscopy and µ-FT-IR.	[8]
Sewage sludge		Wet sludge (314 ± 145 particles L^−1^) fragment length: 36–377 µm fibers length: 213–4716 µm Dry sludge (302 ± 83 particles L^−1^) fragment length: 29–533 µm fibers length: 71–2224 µm	30 mL of H_2_O_2_ (33%) was added to 1 g of sludge (at 50 °C).		
Sewage sludge	China	5553–13,460 particles kg^−1^ Common polymers: PET, PE, and polyacrylonitrile	Samples were dried, sieved, and predigested with 30% H_2_O_2_ at 70 °C.	Three flotation solutions (NaI, ZnCl_2_, and NaCl) with respective densities of 1.8, 1.5, and 1.2 g cm^−3^ were compared. Four membrane types (quartz, glass fiber, PTFE, and nylon) were tested for vacuum filtration of the supernatant. MPs on the membranes were digested with 30% H_2_O_2_ and H_2_SO_4_ at 70 °C for identification using stereomicroscopy and µ-FT-IR.	[81]
Soil		420–1290 particles kg^−1^ Common polymers: PP, PE			
Alluvial soil	Slovenia	Recovery rate: Oil-based extraction: >97% for all vessels in alluvial soil; 80% for modified syringes in compost. Salt-based extraction: >93% and >98% for alluvial soil and compost, respectively The ZnCl_2_ solution could be reused up to 20 times without losing the desired density, reducing cost and environmental impact. Protocol was chosen to extract PE, PP, PS, PVC, and PET at various concentrations (10, 25, and 50 MPs) with cumulative recoveries >93% for all spiked MPs.	-	Oil-based extraction: 30 mL of H_2_O and 3 mL of olive oil were added to the sample. The vessel was capped and shaken. After 2 h of standing, the samples were frozen overnight at −18 °C. The frozen oil layer was removed, melted, and filtered through a GF/C filter (47 mm). The MPs were rinsed with n-hexane and H_2_O. Salt-based extraction: ZnCl_2_ solution (1.6 g cm^−3^; pH 3) was filtered with GF/C filter. The sample was transferred to 50 mL centrifuge tube and a ZnCl_2_ solution was added to the 50 mL mark. After shaking for 30 s, it was centrifuged at 9000 rpm for 15 min. The supernatant was filtered through a GF/C filter. For compost, an oxidation step was performed by submerging the filter in 60 mL of Fenton’s reagent for 2 h under constant stirring. The oxidized sample was filtered through a GF/C filter. The optimal extraction method was tested on samples spiked with 10, 25, and 50 MPs. Identification of MPs via HS-SPME-GC-MS.	[87]
Biowaste compost			10 g of compost with MPs (10 and 20 MPs of LDPE or PET) was added to 60 mL of Fenton’s reagent and left for 2 h under constant stirring. The sample was filtered through the GF/C filter, and the sample remaining on the filter was oxidized using 50 mL of NaOH (1M) under constant stirring overnight at 50 °C. The sample was filtered through a 100 µm stainless-steel mesh.		
Lagooning sludge	Morocco	Fresh sludge: 40.5 ± 11.9 × 10^3^ kg^−1^ (dw) Dewatered sludge: 36 ± 9.7 × 10^3^ kg^−1^ (dw). Sludge dewatering resulted in a loss of MPs <500 μm. The quantity of MPs in compost varied with the proportion of sewage sludge.	20 g of dry sample treated with Fenton’s reagent (H_2_O_2_ 30% (*v*/*v*) + FeSO_4_ solution 20 mg mL^−1^).	For Py-GC/MS analysis, 1 mg of the freeze-dried sample was used. For Nile red staining, 1 g of digested sample was mixed with 50 μL Nile red (1 mg mL^−1^) and incubated on a shaker at 100 rpm for 60 min. MPs were separated using a ZnCl_2_ solution (1.38 g cm^−1^) and centrifuged. The supernatant was filtered through 0.45 μm cellulose nitrate filters with a graduated grid (100 μm). The filter paper was observed using a fluorescence microscope under blue light. Identification of suspected MPs using Raman spectroscopy	[112]
Soil Sediment Compost Sewage sludge Suspended particles of WWTP Indoor dust	Germany	Recoveries rates for PET: 94.5–107.1% Limit of determination 0.031 mg PET Limit of quantification 0.121 mg PET PET mass contents in environmental samples: <LOQ in agriculture soil up to 57,000 mg kg^−1^ in dust samples.		20 g of sample was mixed with 20 mL of 1-butanol and 1 g of potassium hydroxide pellets. The mixture was heated to 115 °C in an oil bath under constant stirring for 1 h. Then, 50 mL of ultrapure water was added, and the system was mixed for 1 h at 300 rpm. The extract was vacuum-filtrated using a glass-fiber filter. Next, 10 mL of the aqueous phase was diluted (1:10) with ultrapure water, and the pH was adjusted to 2.5, using HCl (10%). Determination of the monomers, terephthalic acid, using LC-UV. Mass content verification using TED-GC-MS.	[110]
Biosolids	Ireland	4196–15,385 particles kg^−1^ dw. Common polymers: HDPE, PE, PET, PP, and PA. Common types: fibers (75.8%), fragments, films, and spheres	Biosolids treated by composting or thermal drying (TD): samples were soaked in water for 1 week, then transferred to a water bath (30 °C) for 24 h and shacked for 12 h. The samples were sieved through a 250 μm mesh. Biosolids treated by lime stabilization (LS) and biosolids treated by anaerobic digestion (AD): samples were soaked in tap water and washed through 250, 212, 63, and 45 μm sieves.	TD and AD Extraction: each sample (40 g) was elutriated. The extracted mixture was filtered through a 250 μm mesh, rinsed with ZnCl_2_ (1 M), and brought to a volume of 300 mL. The mixture was shaken (1 min) and settled (20 min). The settled material was drained, and the remaining sample was filtered (glass-fiber filter).LS Extraction: 10 g from each sample was filtered. Identification: Stereomicroscopy, ATR-FT-IR, and SEM.	[57]
Compost	Finland	Mean recovery rate: 90.5% for PE, PU, polycarbonate (PC), PET, PVC, and PS; 30% for tire wear particles (TWP). Sewage sludge compost: 2 acrylonitrile (butadiene styrene) (ABS) fragments, and 1 PE fiber. Biowaste compost: 1 PE fragment, 1 PET fiber, 1 ABS fragment and 1 blend ABS/PET fiber.	10 mL H_2_O_2_ (30%), 1 mL of FeSO_4_.7H_2_O (2 mmol L^−1^), 1 mL of protocatechuic acid (2 mmol L^−1^), and 5 mL of H_2_O were added to 1 g of soil. Samples were kept in the hood for 1 h and then in the oven (40 °C) overnight.	Oxidized samples were transferred to custom-made PTFE tubes with 3 mL of olive oil, left to stand for 2 h, and frozen at −40 °C. Samples were filtered using glass microfiber filters. Filters were rinsed with water and n-hexane, air dried, and the MPs were collected with tweezers for identification with ATR-FT-IR. Each sample was extracted three times.	[84]
Agriculture soil		Mean recovery rate: 96.5% for PE, PU, PC, PET, PVC, and PS; 43% for TWP. 1 ABS fiber and PS fragment detected in soil from Mäkelä.			
Amended agricultural soil	Chile	18–41 particles g^−1^ Common types: microfibers	Samples were milled (porcelain mortar), sieved (<1 mm), and dried.	5 g of each sample was mixed with 20 mL of deionized water, centrifuged, and filtered with filter paper. 20 mL of NaCl was added to the sample, which was centrifuged and filtered with filter paper. 20 mL of ZnCl_2_ was added, centrifuged, and filtered with filter paper. MPs identified by stereomicroscopy.	[70]
Agricultural soil	Chai river valley, China	0–5 cm layer: Fibers: 11,130–24,850 particles kg^−1^ Strings: 10–60 particles kg^−1^ Films: 460–1110 particles kg^−1^ fragments: 230–1360 particles kg^−1^ 5–10 cm layer: Fibers: 9780–26,940 particles kg^−1^ Strings: 40–70 particles kg^−1^ Films: 410–1290 particles kg^−1^ Fragments: 170–15,700 particles kg^−1^	10 mL of H_2_O_2_ (35%) was added incrementally to 30.0 g of soil sample. FeSO_4_ (10%, 1 mL) was added, and sample was heated on sand bath (50 °C). After organic matter destruction, FeSO_4_ (10%, 1 mL) and NaOH (0.5 M, 30 mL) were added. Volumes were adjusted to 150 mL with distilled water, sonicated, and centrifuged.	Supernatant was collected and 150 mL of a saturated NaI solution (1.8 g cm^−3^) was added to the soil, followed by centrifugation. The procedure was repeated. The collected supernatant was filtered (1, 0.25, and 0.05 mm mesh). H_2_O_2_ (2 mL) was added for the digestion of labile organic. Solids were washed with distilled water, transferred to clean glass containers and oven-dried at 80 °C. MPs were optically sorted by a dissecting microscope.	[28]
Agricultural soil	Middle Franconia, Germany	0–1.25 particles kg^−1^ dw 16 particles per 50 kg dw (5–1 mm): PE (62.50%, 10 particles) PP (25.00%, 4 particles)	Soil aggregates dissolved by adding 20 mL of H_2_O_2_ to 500 mL of sample. Size fractioning using sieves with mesh size of 5 and 1 mm. The procedure was repeated until all aggregates were dissolved.	MPs optically sorted under a magnifying lamp and by stereomicroscopy. Identification and quantification carried out by ATR-FT-IR.	[58]
Soil	United Kingdom	Average recoveries using high-gradient magnetic separation (HGMS) system: 96% for fibers and 92% for MPs in loam; 91% for fibers and 87% for MPs in high-carbon loamy sand; 96% for fibers and 89% for MPs in sandy loam; 97% for fibers; and 94% for MPs in high-clay sandy loam. Agricultural soil: HGMS extracted an average of 14 ± 4 fibers and 3 ± 1 MPs per 8 g sample extracted using HGMS; 8 ± 3 fibers and 1 ± 1 MPs per 8 g sample extracted by density separation.	20 mL of ethanol was added to 4 g of soil spiked with 30 MPs (<1% (*w*/*w*)) which were prepared in the laboratory. Magnetic soil particle removal for each soil sample using HGMS system. MPs magnetized with modified iron nanoparticles.	HGMS: the sample was introduced in the system and the retained fraction containing MPs was filtered onto 1.2 μm Whatman GF/C glass-microfiber membrane filters. Density separation: 50 mL of a saturated zinc bromide solution (density ≈ 2.4 g mL^−3^) was added to the soil. The sample was shaken at 300 rpm for 5 min and settled overnight. The recovered material was treated with 10 mL 30% H_2_O_2_ for 1 h at 60 °C and filtered. The MPs were counted directly on the filter using a stereo microscope. ATR-FT-IR was used to confirm the compositions of the MPs.	[98]
Loamy sand (standard)	Germany	LOD: 0.07 wt % PET; LOQ: 1.72 wt % PET. MS signal intensities linearly responding to MPs concentrations.	Samples (with 1.61 ± 0.15 wt % organic matter) spiked with 0.23–4.59 wt % PET recycled MPs.	Calibration series was prepared with a standard loamy sand with 1.61 ± 0.15% organic content. DL-cysteine was used as the internal standard. PET quantified by TGA-MS.	[114]
Soil (standard)		Validation parameters for plastic contents of 250 μg g^−1^: R^2^ > 0.996, LOD: 1–86 ng Precision: 3.2–7.2% Recoveries: 70–128% The addition of nontarget polymers (PET, PVC and TWP) did not interfere with the quantification of the analytes.	Each reference soil (4 g) was spiked with 0.2 and 1.0 mg of PE, PP, and PS. Three clean-up protocols were tested: (1) adding 8 mL of methanol to the sample and agitating it for 60 min at 150 rpm, followed by centrifugation and evaporation of the supernatant; (2) performing Fenton digestion by adding 10 mL of FeSO_4_.7H_2_O solution (20 g L^−1^, pH 2) and 10 mL of H_2_O_2_ (30%) to the spiked soil, followed by 60 min in an ice bath and heating to 60 °C; (3) adding 4 mL of KAl(SO_4_)_2_.12H_2_O solution (500 mgL^−1^) to the soil, shaking for 60 min at 150 rpm and evaporation. Nonspiked soil and soil spiked with 0.2 mg of nontargeted plastics were also included for testing	Polymers extracted using 1,2,4-trichlorobenzene (TCB) and liquid sample aliquots analyzed by Py-GC-MS.	[115]
Road Dust	Korea	Tire and road-wear microparticles (TRWMPs): 6,400–39,738 μg/g. Average concentration of TRWMPs in the industrial area of 22,581 μg/g and in the residential area of 9054 μg/g.	Moisture content was removed at 120 °C.	TGA to compare the thermal profile of road dust and tire tread particles; EGA-MS analysis to compare the organic composition of road dust and tire tread powder; identification and quantification of TRWMPs using Py-GC-MS.	[77]

## 5. (Eco)Toxicological Effects of Microplastics

Considering the presence of MPs in the environment (Table 1), it is very likely that terrestrial and aquatic organisms can find and interact with MP particles present in their ecosystems [2]. Studies have suggested that MPs can be ingested by fish [10,11,12,13,14,15,16,17], crustaceans [38,116,117], nematodes [16], arthropods [118], and annelids [25,119,120], or can be absorbed by plants such as *Lolium perenne* [25], *Allium fistulosum* [121], and *Lepidium sativum* [122] (Table 2). The great challenge in assessing the toxicological effects of MPs derives from the fact that MPs are not homogeneous but rather a mixture of particles of different shapes, sizes, and compositions, which can act as vehicles for the transfer of additives and other chemical contaminants [116,123]. Most additives are attached to the polymer matrix via weak van der Waals forces, which means that they can leach from the matrix and become available for uptake (ingestion/sorption) [24,38,124]. PS microspheres are often used as model particles in toxicological tests because PS is one of the most produced polymers and is commonly found in the environment [116]. However, other polymers that contain many additives, such as PVC, are also abundant in the environment and need to be considered [1,18]. 

**Figure 4:**
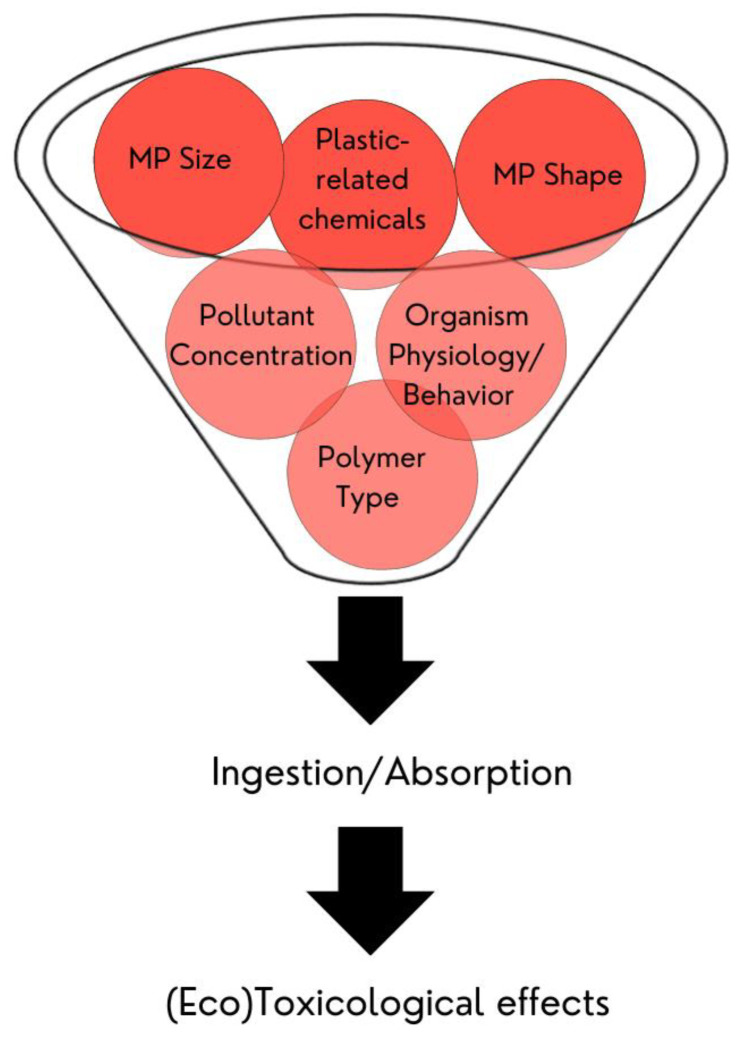
Factors that lead to the ingestion or absorption of microplastics causing potential ecotoxicological and toxicological impacts.

The ingestion of MPs is closely related to the size and shape of the particle, type of polymer, concentration, and physiological and behavioral characteristics of the organism itself (Figure 4) [15,16,38,119,120]. It is more likely that, in the environment, organisms are more exposed to irregularly shaped secondary MPs, which could potentially cause more injury and stronger gut inflammation than would spherical shapes [10,11,13,38]. Additionally, depending on their size, MPs and/or their chemical additives can pass through the intestinal wall and reach other body tissues, which may result in trophic transfers into and up the food chain [2,12,116]. The accumulation of MPs in the gills, gut, and liver may cause obstruction, physical damage, histological changes in the intestines, changes in lipid metabolism, liver metastasis, behavioral changes, growth and developmental inhibition, endocrine disruption, energy disturbance, oxidative stress, immune and neurotransmission dysfunction, and genotoxicity [10,11,13,15]. Even if plastic is excreted, there is evidence suggesting that retention time in the intestine is longer than that observed for food [2].

In a study from Choi et al., the effects of irregular and spherical PE microparticles on swimming behavior, gene expression, and *Cyprinodon variegatus* larvae were compared [10]. No mortality or malformations were observed in the larvae; however, there was a decrease in swimming activity that was more pronounced in the presence of irregular MPs. In addition, the MPs ingested by *C. variegatus* passed into the digestive system, causing a distension of the intestinal lumen. Exposure to both shapes of MPs slightly increased cellular reactive oxygen species (ROS), causing a differential regulation of genes related to oxidative stress (*Cat* and *Sod3*). *Sod3* gene expression was induced for both shapes at 50 mg L^−1^, whereas *Cat* gene expression was induced at 250 mg L^−1^ of spherical MPs and at 50 mg L^−1^ of irregular MPs. Moreover, catalase (CAT) activity, an antioxidant-related enzyme, significantly increased after exposure to 50 mg L^−1^ of irregular MPs [10]. The influence of MPs’ shape was also studied in *Carassius auratus* by Jabeen et al. [11]. The fish were exposed to ethylene vinyl acetate (EVA) fibers, PS fragments, and PA pellets via food amended with MPs for six weeks, and no mortality was observed. However, the organism showed a significant weight loss, and fibers were found in the gills, gastrointestinal tract, and feces. The fragments and pellets were chewed, and then expelled, and a histological examination revealed a breakage of the dermal layer with hemorrhages in the lower jaws. Moreover, although MPs did not accumulate in the organs of *C. auratus*, changes in liver tissue (passive hyperemia, dilated sinusoid, and hydrophilic vacuolization) were detected. They may be related to the stress induced by the chemical additives released during chewing and ingestion [11]. Similarly, Qiao et al. reported that the shapes of PS microparticles had a significant influence on their uptake by *Dania rerio* [13]. The study suggested that all shapes accumulated in the gut with the following order of magnitude: fibers (8.0 μg mg^−1^) > fragments (1.7 μg mg^−1^) > spheres (0.5 μg mg^−1^), causing damage to the mucosa and an increased permeability, inflammation, and interruption of metabolism. Furthermore, fibers induced a higher activity of superoxide dismutase (SOD) (antioxidant-related enzyme), which may be due to the long residence time, higher accumulation, and larger physical damage in the gut [13]. 

It is not clear whether an irregular shape is the main condition that promotes the bioaccumulation of MPs. Since there is evidence that some organisms (such as *C. auratus*) select the food based on its morphology, the size of the MPs also seems to influence their accumulation in the organs [11,16]. A study involving *D. rerio* focused on the effects of small-sized MPs in the digestive system and found that exposure to PS microspheres (5 μm) resulted in a significant increase in CAT and SOD activity in the intestine [14]. A histological analysis revealed inflammation, bowel wall thinning, villi formation, and epithelial damage. Disturbances in the gut biota have also been identified: a decreased number of proteobacteria and an increased number of fusobacteria [14]. This microbiota dysbiosis could be associated with the development of metabolic disorders [13,14]. Moreover, in their study, Lei et al., exposed *Caenorhabditis elegans* nematodes to MPs of PS with different sizes (ranging from 0.1 to 5.0 μm) and to MPs of PA, PE, PP, and PVC, all of which were approximately 70 μm in size [16]. They found that PS particles measuring 1 μm were the most abundant in the digestive system, causing oxidative stress and pronounced alterations in intestinal calcium levels. 

A study by Zimmermann et al. compared the effects of different types of MP (PVC, PUR, and polylactic acid (PLA)) and their additives on the mortality, reproduction, and body lengths of *Daphnia magna* [38]. All MP particles had irregular shapes and rough surfaces and were identified in the gastrointestinal tract of *D. magna*; however, only exposure to PLA increased mortality. The mean body length of adults was significantly lower when exposed to PVC, PUR, and PLA, whereas reproduction was affected and significantly reduced by exposure to PLA and PUR microparticles [38]. Regarding the contribution of plastic additives to MPs’ toxicity, the study showed that chemical additives leached from PVC were the main driver for the toxicity induced in *D. magna*, reinforcing the idea that toxicological effects can be mediated by both particles and chemicals [38]. In addition, this study highlights that biopolymers (such as PLA) are not necessarily less toxic to organisms than fossil-based polymers, a perspective that is also shared by Boots et al. in a study with perennial ryegrass (*L. perenne*) [25]. 

Given the importance of soil biota in maintaining the structure and function of soil ecosystems, it is essential to investigate the impact of MPs on these organisms [25,119,125]. Studies reporting the exposure and ecological effects of MPs in soil are emerging, as it is recognized as a primary source of MP pollution [25,122]. Earthworms, considered ecosystem engineers, are widely used in ecotoxicological tests due to their sensitivity to toxic substances [25,119]. In a study by Jiang et al., *Eisenia fetida* exposed to concentrations of 10–1000 μg kg^−1^ of PS fragments (100–1300 nm) presented damage to intestinal cells and changes in the activity of antioxidant enzymes glutathione (GSH) and SOD [119]. Moreover, the exposure to 1300 nm MPs at 1000 μg kg^−1^ resulted in the highest accumulation rate. The comet assay revealed that DNA damage in coelomocytes (phagocytic leukocytes) depends on the size and concentration of MPs, and *E. fetida* exposed to a concentration of 1000 μg kg^−1^ with a size of 1300 nm experienced the greatest damage to their DNA [119]. Another study found that exposure to PVC microparticles significantly inhibited the growth and reproduction of springtail *Folsomia candida* by 16.8 and 28.8%, respectively, while also altering the microbiota of the collembolan gut. Higher values of nitrogen and carbon isotopes (δ15N and δ13C) were observed in the tissues, and this alteration may be due to the response of growth rate and metabolic turnover [118]. Likewise, Selonen et al. observed that microsized tire particles (<180 μm) spiked in soil or food reduced reproduction and survival rates and a decrease in the acetylcholinesterase activity of soil invertebrate species (enchytraeid worm *Enchytraeus crypticus*, *F. candida*, and woodlouse *Porcellio scaber*) [65]. Moreover, tire-particle-related chemicals such as benzothiazole and zinc act as neuroinhibitory, affecting the neurological processes of *P. scaber* by reducing the acetylcholinesterase activity [65]. These observations indicate that even at low concentrations, MPs can be ingested by soil organisms and potentially affect their survival and metabolic processes, highlighting the potential for accumulation/magnification of MPs along the soil detrital food web [16,27]. These accumulated substances may also be transferred to predators such as insects, birds, and small mammals [126,127,128]. For instance, Kwon et al. found that oral exposure to PS-MPs can lead to their accumulation in the microglial cells of the mouse brain, causing morphological changes and potentially leading to cell death [125]. These findings are particularly significant because they suggest that PS-MPs can cross the blood–brain barrier in humans affecting the immune response in the central nervous system. 

While studies on the impacts of MPs in soil systems have mainly focused on animals, the effects of MPs on plants have also been observed; however, the number of studies on this topic remains relatively limited [32,129]. MPs can have both direct and indirect harmful effects on plants. Indirect effects include alterations to soil structure, which can impact root growth, nutrient immobilization, shifts in soil microbial communities, and root symbionts, potentially leading to reduced fertility and contaminant transportation and leaching [32,66,129]. The direct effects of MPs on plants include the clogging of seed pores, reduction in germination rates, and accumulation in plant tissues [32,129]. It is important to note that different plants can be affected by MPs to differing degrees with different mechanisms and effects [32]. Moreover, terrestrial plants are temporary sinks of atmospheric MPs. In the top 11 green countries, there could be 0.13 trillion pieces of MPs attached to leaves. Though there is no direct evidence of impaired plant function at environmental concentrations, the long-term ecological effects of MPs should be investigated [130]. 

Pignattelli et al. investigated how acute (6 days) and chronic (21 days) exposure to four different types of MPs (PP, PE, PVC, and PE + PVC) affected the growth and biochemical responses of garden cress (*L. sativum*) [122]. The study found that acute exposure to PE had adverse effects on the germination rate, plant height, leaf number, and fresh biomass production. On the other hand, chronic exposure to PP and PE negatively impacted germination rate, leaf number, and biomass produced. Moreover, PVC was found to be the most toxic MP with the highest production of H_2_O_2_ and depletion of ascorbic acid (AsA), suggesting that different MPs act in different ways [122]. In other study the impact of sewage sludge containing MPs on the growth, biomass production, and yield of tomato plants (*Lycopersicon esculentum* Mill.) was examined over a period of 109 days. Plants grown in soils mixed with sludge showed a more pronounced growth while fruit production was delayed and reduced [131]. These findings suggest that plants are vulnerable to the impacts of MPs, although more research is needed to fully understand whether the effects on terrestrial plants are due to the MPs themselves, their chemical-related contaminants, or both. What about the effects of NPs on terrestrial plants? Li et al. studied the effects of PS-NPs (100–700 nm) on *Cucumis sativus* growth using Hoagland nutrient solution [132]. The results showed that different sizes of PS-NPs had varying effects on plant metabolic pathways: 700 nm PS-NPs increased the levels of the oxidative stress markers in leaves, such as malondialdehyde (MDA), proline, and H_2_O_2_; and 100 nm PS-NPs decreased chlorophyll and soluble sugar contents in the leaves. Additionally, the study found that the degradation of PS-NPs into benzene rings in cucumber leaves may be the main factor affecting chlorophyll and sugar metabolism, suggesting harmful effects on plant growth and development. Nevertheless, further research in this field is necessary [132]. 

It is possible that MPs could contaminate human food and therefore enter the human diet. In fact, plastic fragments have been found in a range of food sources, including seafood, bottled water, salt, honey, edible fruits and vegetables, and even infant milk powder [127,133,134,135,136,137,138]. Additionally, airborne particles with sizes ranging from 1 nm and 20 µm can also be inhaled by humans [139,140]. Plastic fragments have been found on both the maternal and fetal sides of human placentas, suggesting that exposure to plastic debris may occur during fetal development [141]. Moreover, studies have shown that certain types of MPs, such as PS-MPs and PE-MPs, can cause alterations to the shape of lung cells, slowing down their metabolism [142], and causing genomic instabilities in human peripheral lymphocytes [143], respectively. These findings suggest that MPs that humans encounter via ingestion or inhalation can potentially enter the bloodstream, become bioavailable, and translocate to organs [140]. Therefore, it is critical to conduct further research to fully understand the fate and transport mechanisms of MPs in the environment, their ability to sorb and transfer organic and inorganic contaminants, and the potential long-term effects of exposure. This is essential for conducting effective risk assessments and developing strategies to mitigate the potential impact of MPs on human health. 

**Table 2:** Illustrative toxicological effects observed in diverse organisms, including fishes, crustaceans, nematodes, arthropods, and annelids, and plants, caused by MPs of varying shapes, types, and sizes.

Species	Exposure Characteristics	Toxicological Effects/Findings	Ref.
*Artemia salina*	PS (spherical; 5 μm); 1–100 mg L^−1^; 48 h and 14-day exposure tests	Deformation of epithelial cells in the midgut region after both acute exposures at 100 mg/L and chronic exposure at 1 mg L^−1^	[117]
*Carassius auratus*	EVA (fiber; 0.7–5.0 mm), PS (fragments; 2.5–3.0 mm), PA (pellet; 4.9–5.0 mm); 6 weeks of exposure	Fibers detected in gills, gastrointestinal tract and feces; severe alterations in the livers of fish exposed to fibers; severe breakage of the dermal layer with hemorrhages in the lower jaws of fish exposed to fragments; hypertrophy of mucous cells in the lower jaw in fish exposed to pellet.	[11]
*Cyprinodon variegatus*	PE (spherical, 150–180 µm; irregular, 6–350 µm); 50 and 250 mg L^−1^; 4-day exposure test	Intestinal distention provoked by the accumulation of MPs in the digestive system; decrease in swimming activities; oxidative stress induced by irregularly shaped MPs.	[10]
*Danio rerio*	PS (fibers, fragments, beads); 50–500 μg L^−1^; 21-day exposure test	Shape-dependent accumulation in the gut: fibers > fragments > beads; mucosal damage and increased permeability; inflammation and metabolism disruption; gut microbiota dysbiosis and bacteria alterations.	[13]
	PS (spherical; 5 μm); 50–500 μg L^−1^; 21-day exposure test	Accumulation of MPs in the gut; inflammation and oxidative stress in the gut tissues; gut microbiome perturbations: *Proteobacteria* decreased and *Fusobacteria* increased.	[14]
	PS (spherical; 50–500 nm); 0.1–10 mg L^−1^; 14-day exposure test	Tissue of the amputated plane was penetrable by MPs; inhibition of fin regeneration, both morphologically and functionally, of amputated larvae.	[15]
	PA, PE, PP, PVC (~70 μm); PS (0.1–5.0 μm); 0.001–10.0 mg L^−1^; 10-day exposure test	PA, PE, PP, and PVC caused intestinal damage including cracking of villi and splitting of enterocytes.	[16]
	MPs: PS (700 nm)	Signs of accumulation of particles around the heart region and within the blood stream; systemic immune responses; lipid metabolism and toxicity pathway significantly enriched.	[17]
	Fertilized eggs exposed to MPs (2 mg/L, red fluorescent spherical polymer particles), copper (Cu), and Cu + MPs, 96 h exposure	MPs did not significantly impact the early life stages of zebrafish; increased mortality, inhibition hatching rate, oxidative stress, AChE inhibition, and behavioral changes in zebrafish in the presence of Cu; antagonistic response of MPs to Cu.	[144]
*Dicentrachus labrax*	Mixture of environmental MPs (35.29% PEVA; 5.88% HDPE; 17.65% PE; 11.76% LDPE; 23.53% PA; 5.88% PP); fragments and fibers (5–1 mm and 1 mm–300 µm)	Imbalance in the enzymatic defense mechanisms after a short-term exposure to MPs ranging from 1 mm to 300 µm.	[47]
*Mytilus edulis*	PLA (<250 μm); 10 μg/L and 100 μg L^−1^ 8-day exposure test	No significant signs of oxidative stress or neurotoxicity; slight increase in CAT and glutathione-S-Transferase (GST) biomarker activities was observed.	[145]
*Sparus aurata* L.	PVC (40–150 μm); 100 and 500 mg kg^−1^; 30-day exposure test	Ingestion did not produce any significant alteration in humoral and cellular immunity; evidence of cellular and oxidative stress and damage in the liver and kidney.	[12]
*Daphnia magna*	PVC, PUR and PLA (<59 µm); 10–500 mg L^−1^	PVC toxic to reproduction; PLA reduced survival.	[38]
	PS (6 µm); 5–300 mg L^−1^; 120 h and 80-day exposure tests	EC_50_ of 34.3 (19.8–59.3) mg L^−1^ for juveniles and 52 (17.7–152.3) mg L^−1^; growth rate of mother animals and the body size of newborn declined with increasing dose of MPs.	[116]
*Caenorhabditis elegans*	PA, PE, PP, PVC (~70 μm); PS (0.1–5.0 μm); 0.5–10.0 mg m^−2^; 2-day exposure test	5.0 mg m^−2^ significantly inhibited survival rates, body length, and reproduction; oxidative stress and changes in intestinal calcium levels.	[16]^]^
*Coturnix japonica*	Naturally aged PS (fragments); 9-day oral exposure tests; three experimental groups (0, 11, and 22 MPs per day)	Lower body mass; increased ROS levels in muscle and liver, and higher production of MDA in various organs; reduction in SOD activity in the liver and intestine; MPs’ size and shape altered as they moved through the gastrointestinal tract.	[146]
*Eisenia andrei*	Tire particles (<600 µm) and PS (<500 µm, containing 1% hexabromocyclododecane (HBCD)); 28-day exposure test	No mortality, morphological, or avoidance behavior changes observed; AChE activity was not significantly affected after 14 and 28 days.	[147]
*Eisenia fetida*	PS (fragments, 100–1300 nm); 100–1000 μg kg^−1^; 14-day exposure test	Intestinal cells were damaged; changes in GSH level and SOD activity; induction of damages in DNA.	[119]
*Enchytraeus crypticus*	PA and PVC (13–18 μm; 90–150 μm); 20–120 g kg^−1^; 21-day exposure test	EC_50_ of 108 ± 8.5 g kg^−1^ for PA 13–18 μm size range; 25% reduction in reproduction for PA 90–50 μm.	[120]
*Folsomia Candida*	PVC (80–250 μm); 1 g Kg^−1^ dw; 56-day exposure test	Alterations of microbial community of the gut; significant reduction in body weight and reproduction; carbon and nitrogen contents increased in tissues.	[118]
*Aporrectodea rosea*	PLA, HDPE and fibers	The biomass of *A. rosea* exposed to HDPE was significantly reduced.	[25]
*Allium fistulosum*	PE (fibers); PA (spherical); PE, PS, Polyester, and PET (fragments)	Changes in biomass, tissue elemental composition, root traits, and soil microbial activities.	[121]
*Cucumis sativus* L.	PS-NPs (100, 300, 500, and 700 nm); 10 mg mL^−1^, 65-day exposure test	Decreased biomass induced by 300 nm PS-NPs; decreased chlorophyll content and soluble sugar induced by 300 nm PS-NPs; increase in the contents of MDA and proline in leaves induced by 700 nm PS-NPs.	[132]
*Lepidium sativum*	PP, PE, PVC, PE + PVC (0.125 mm); 184 ± 4 mg kg^−1^	Effect on growth; induction of oxidative stress.	[122]
*Lycopersicon esculentum Mill*	One-month-old plants transplanted outdoors into 3.3 L terracotta pots with five different soil compositions: control, manure control, and soil + sewage sludge 109-days exposure test	Lower crop in plants grown under soil mixed with sewage sludge.	[131]
*Lolium perenne*	PLA, HDPE and fibers	Germination decreased with PE and fibers; reduction in shoot height with PLA; increase in chlorophyll a/chlorophyll b ratio suggesting a stronger inhibition of chlorophyll b synthesis in response to MPs.	[25]
*Oryza sativa*	PS and PTFE (~10 mm) combined with arsenic (As) III in Hoagland nutrient solution; 0.04 g L^−1^ MPs + 1.6 mg L^−1^ As(III); 0.1 g L^−1^ MPs + 3.2 mg L ^−1^ As (III); and 0.2 g L^−1^ MPs + 4.0 mg L ^−1^ As (III)	PS and PTFE hinder root growth and transpiration; As (III) damages chloroplast structure, reducing photosynthetic capacity and biomass, and impairing antioxidant enzyme structure, leading to membrane lipid peroxidation and membrane structure destruction; the presence of MPs restricts the uptake of As(III) in rice seedlings, reducing its content in tissues.	[148]
*Triticum aestivum*	LDPE, PET, PBT and starch-based biodegradable MP	Biodegradable MPs inhibited wheat growth and decreased the fruits and the shoot biomass.	[149]
*Vicia faba*	PS (5 μm and 100 nm); 10–100 mg L^−1^; 48 h exposure test	Inhibition of growth; induction of oxidative damage; accumulation of 100 nm sized MPs in root.	[150]

## 6. What Does the Future Hold?

Plastic pollution has gained attention due to the accumulation of waste in natural ecosystems and the breakdown of plastics into MPs via physical, chemical, and biological drivers. Identifying the sources of MPs in the environment is challenging due to factors such as a high plastic production and consumption, leakage from waste streams, and polymer heterogeneity. The distribution and pathways of MPs in ecosystems remain unsuccessfully understood. Toxicological tests have revealed that MPs can be ingested by a variety of organisms, including, but not limited to, fish, crustaceans, nematodes, arthropods, and annelids. There is evidence that MPs accumulate in the gills, gut, and liver of these organisms, causing gut obstruction, behavioral changes, and physical and histological damage. Moreover, MPs can enter the food chain, with potential consequences for human and environmental health. The shape, size, and concentration, composition of MPs as well as the characteristics of the organism and the environment in which they are found may contribute to the observed morphological and toxicological effects. 

Assessing the toxicity of MPs is challenging due to their heterogeneous nature and the fact that they can act as carriers for other chemicals that they absorb from the surrounding media. Future studies should focus on the effects of a realistic mixture of polymers, using environmentally relevant concentrations, to understand the extent effects of MPs. The field is claiming standardization may enable a direct comparison between studies and the use of environmentally relevant exposure concentrations in (eco)toxicological assays. However, several key challenges must be overcome to achieve this goal. These challenges include the absence of reference materials for method validation, the use of nonstandardized analytical methods, and difficulties associated with complex matrices rich in organic matter. 

The future of MP research involves further investigation into the long-term effects of MPs on the environment and human health. Additionally, there is a need for increased interdisciplinary collaboration between scientists, policymakers, and industry leaders to address the issue of MP pollution comprehensively. The world population is expected to increase in the coming decades, which will translate into increased environmental pressures, consumption of raw materials, and waste generated. It is naive to think that plastic pollution will be solved with the transition to bioplastics and biobased plastics, whose environmental impacts of production (e.g., use of land and water resources) are still under discussion. Additionally, biobased plastics can still generate waste and MPs, which are not proven to be less dangerous than oil-based MPs. Tackling plastic pollution requires a comprehensive and holistic approach that includes not only the production and disposal aspects but also focuses on consumption patterns concerning single-use plastics and explores potential methods to minimize waste generation. 

## Data Availability

Not applicable.
